# VRK1 regulates Cajal body dynamics and protects coilin from proteasomal degradation in cell cycle

**DOI:** 10.1038/srep10543

**Published:** 2015-06-12

**Authors:** Lara Cantarero, Marta Sanz-García, Hadar Vinograd-Byk, Paul Renbaum, Ephrat Levy-Lahad, Pedro A. Lazo

**Affiliations:** 1 Experimental Therapeutics and Translational Oncology Program, Instituto de Biología Molecular y Celular del Cáncer, Consejo Superior de Investigaciones Científicas (CSIC), Universidad de Salamanca, Salamanca, Spain; 2 Instituto de Investigación Biomédica de Salamanca (IBSAL), Hospital Universitario de Salamanca, Salamanca, Spain; 3 Medical Genetics Institute, Shaare-Zedek Medical Center, Hebrew University of Jerusalem Medical School, Jerusalem, Israel

## Abstract

Cajal bodies (CBs) are nuclear organelles associated with ribonucleoprotein functions and RNA maturation. CBs are assembled on coilin, its main scaffold protein, in a cell cycle dependent manner. The Ser-Thr VRK1 (vaccinia-related kinase 1) kinase, whose activity is also cell cycle regulated, interacts with and phosphorylates coilin regulating assembly of CBs. Coilin phosphorylation is not necessary for its interaction with VRK1, but it occurs in mitosis and regulates coilin stability. Knockdown of VRK1 or VRK1 inactivation by serum deprivation causes a loss of coilin phosphorylation in Ser184 and of CBs formation, which are rescued with an active VRK1, but not by kinase-dead VRK1. The phosphorylation of coilin in Ser184 occurs during mitosis before assembly of CBs. Loss of coilin phosphorylation results in disintegration of CBs, and of coilin degradation that is prevented by proteasome inhibitors. After depletion of VRK1, coilin is ubiquitinated in nuclei, which is partly mediated by mdm2, but its proteasomal degradation occurs in cytosol and is prevented by blocking its nuclear export. We conclude that VRK1 is a novel regulator of CBs dynamics and stability in cell cycle by protecting coilin from ubiquitination and degradation in the proteasome, and propose a model of CB dynamics.

Cajal bodies (CBs), discovered by Ramón y Cajal in 1903^1^,^2^, are dynamic nuclear organelles without a membrane and that are enriched in several nuclear proteins and RNA-protein complexes. However, signalling pathways controlling CBs organization and function are not known. CBs play an important role in RNA processing^3^, particularly those associated with splicing^4^, and assembly and maturation of small nuclear RNPs^5^. CBs contain specific RNAs, such as sca-RNA affecting its biogenesis^6^ and binding to WD40-containing proteins^7^,^8^. Coilin is also associated to telomerase RNA biogenesis^9^ and to processing of U snRNA^10^. Proteins within CBs interact with snRNA, snoRNA^11^ and also with telomerase components^12^,^13^,^14^, where they might also play a regulatory role not yet understood^15^. The interaction of coilin with RNA is also regulated by phosphorylation^16^. These protein and RNA complexes located within CBs are maintained by multiple weak interactions, and CBs components can exchange in a very dynamic way between this organelle and the nucleoplasm^17^. Structurally, CBs are organized and assembled on coilin that plays a scaffold role and is the main structural constituent of CBs^18^,^19^, but coilin is also present in smaller Histone-locus Bodies (HLB)^15^. Coilin is an 80 kDa (p80) protein that self-associates to form CBs^20^, but how this aggregation is regulated in proliferating cells is not known. In addition, CBs contain multiple proteins whose function is not well known^21^,^22^.

Cajal bodies have a dynamic structure during cell cycle, in which they assemble and reassemble^23^ by a regulatory mechanism that is not known. CBs number and size are maximal at G1/ S, but are absent in mitosis and in arrested cells^18^,^24^. Thus, CBs dynamic assembly and disassembly requires regulatory mechanisms that are not yet identified, but in which protein phosphorylation is very likely to play an important role. Coilin undergoes several posttranslational modifications, and its dynamic changes are likely to be regulated by these covalent modifications, including phosphorylations^25^,^26^ and methylations^27^. Coilin is a hyperphosphorylated protein^4^,^25^,^28^,^29^,^30^ in cell proliferation^26^. However, coilin protein levels remain constant throughout cell cycle progression^31^. These coilin phosphorylations are likely to regulate its interaction with other proteins^25^, and thus contribute to different functional roles depending on the particular phosphorylated residue and the interaction partner affected that is affected. Up to now, only two unrelated kinases cdk2/cyclinE complex , which is recruited to assembled CBs^32^, and VRK1^33^ have been shown to directly phosphorylate coilin. VRK1 is a nuclear kinase regulated in cell cycle progression^34^ and might be a candidate to regulate CB dynamic changes in structure and composition in proliferating cells and during cell cycle progression. In cell cycle progression, VRK1 is necessary for the exit of G0 and entry in G1, and its activity and levels increase early in the G1 phase^34^,^35^ reaching its highest level in G2/M and facilitating chromatin condensation^36^. VRK1 is one of the most abundant nuclear Ser-Thr kinases^37^ and hyperphosphorylates human coilin in at least eight residues, including Ser184^33^. Nuclear VRK1 specifically phosphorylates and regulates histone H3^36^,^38^, hnRNP^39^ and several transcription factors including p53^40^,^41^,^42^,^43^, CREB^44^, c-Jun^45^, and ATF2^46^. VRK1 also regulates other mitotic processes^47^, including Golgi fragmentation^48^ and nuclear envelope assembly in mitosis^49^,^50^. In addition VRK1 also interacts with, and regulates, proteins implicated in DNA-damage responses such as 53BP1^51^. The kinase activity of VRK1 can also be inhibited by protein-protein interactions with some nuclear proteins as histones H2A1.2 in interphase^52^, the small GTPase RAN-GDP^38^, and the MKP2 phosphatase^53^. The role of VRK1 in multiple nuclear processes where nucleic-acid-protein complexes are necessary suggests that VRK1 is likely to be an important candidate to play a regulatory role in CB dynamic assembly. Moreover, depletion of coilin^54^,^55^ or VRK1^56^,^57^,^58^,^59^ also partially share a common phenotype of reduced fertility.

In this work we have studied the role that VRK1 plays in the regulation of CBs. We have identified that VRK1 regulates CBs assembly in cell cycle by a specific phosphorylation and also protects coilin from ubiquitin mediated degradation in the proteasome, in which the ubiquitin ligase Mdm2 is implicated. This effect of VRK1 on coilin stability regulates the assembly and disassembly of Cajal bodies in nuclei as well as its variation in cell cycle progression.

## Results

### VRK1 is not present in Cajal bodies but its depletion causes a loss of Cajal bodies

**Figure 1:**
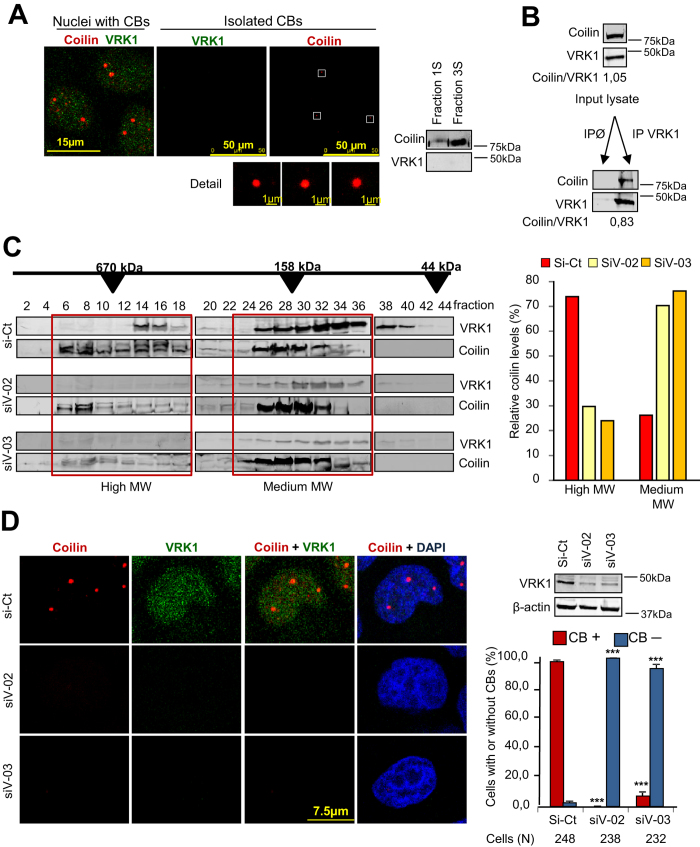
VRK1, coilin and Cajal bodies. **A.** Isolation of CBs containing coilin, but not VRK1. In the immunofluorescence at the left are shown nuclei containing Cajal bodies detected with an anti-coilin antibody. The two images (center and right) show isolated CBs stained for VRK1 (green) or coilin (red). At the bottom are shown magnifications of several individual CBs. In the western blot to the right are shown detection of coilin and VRK1 in CBs (fraction 3S). **B.** Coilin and VRK1 can form an intracellular complex. HeLa cell extract was used for reciprocal immunoprecipitation of human endogenous VRK1 and coilin proteins. The interaction was confirmed using transfected proteins (Fig. S1). **C.** HPLC fractionation of MCF7 cell lysates from si-Control or siVRK1 treated cells. Coilin and VRK1 were detected by immunoblot in individual fractions. The relative value in each fraction (HMW fractions: 6-18); MMW fractions: 26-36) is referred to the total value determined by the sum of all fractions.siCt: si-Control, siV-02: siVRK1-02; siV-03: siVRK1-03; CBs: Cajal bodies. **D.** VRK1 knockdown causes a loss of Cajal bodies. VRK1 was silenced in HeLa cells with two different siRNA. Two different siVRK1 were transfected with Lipofectamine 2000 and seventy-two hours later the presence of coilin in CB by immunofluorescence confocal microscopy as well as VRK1 protein levels was determined (right side blot). Similar experiment was performed in MCF7 (Fig. S1B, S3) and SH-SY5Y cells (Fig. S1B, S4). The number of cells, with and without CBs, was counted with the ImageJ program (graph, right). The distribution of cells, with and without CBs, in different conditions was determined by t-Student. *** (P < 0.0005).

Cajal bodies are present in many cell types and its number ranges from two to six depending on the cell line used (Supplementary Fig. 1A). Because VRK1 is a very abundant nuclear Ser-Thr kinase^37^ we tested whether the VRK1 protein was also present within CBs. For this aim, CBs were isolated from nuclei and the presence of coilin and VRK1 was determined by immunofluorescence and immunoblot (Fig. 1A). Purified CBs were detected by their positivity for coilin (Fig. 1A, detail and immunoblot), but VRK1 was not detected within CBs, thus if VRK1 is present its level must represent a very minor subpopulation. However, a complex between endogenous VRK1 and coilin was detected by immunoprecipitation in lysates from non-synchronized cells (Fig. 1B). This VRK1-coilin stable protein interaction was confirmed using transfected proteins in pulldown and immunoprecipitation assays (Supplementary Fig. S1B, C).

CBs form macromolecular complexes in the nucleus. Therefore, we studied the size of the protein complexes containing coilin and VRK1. For this purpose, cell lysates were prepared under mild conditions and were fractionated by HPLC. Coilin was located in two main fractions of high (700 kDa) and mid (160 kDa) molecular size (Fig.1C). VRK1 was mostly detected in the midsize fraction (Fig.1C), but a minor fraction was also detectable in the high and low MW fractions. However, VRK1 knock-down resulted in the reduction of the coilin protein present in the high molecular size fractions (6 to18) and its increase in the midsize fractions (24–36) (Fig. 1C). This midsize complex may be a basic building block for initiating CBs assembly. These results are consistent with the requirement of VRK1 for facilitating assembly or maintaining coilin within high molecular size complexes.

Coilin is a scaffold protein and it is likely that kinases phosphorylating coilin, such as VRK1^33^, might contribute to the assembly of CBs. Therefore, we determined if VRK1 knock-down could affect the organization of CBs in three cell lines, HeLa (Fig. 1D), MCF7 (Supplementary Fig. S1D, Supplementary Fig. S2) and neuroblastoma SH-SY5Y cells (Supplementary Fig. S1D). In all of them depletion of VRK1 by knockdown resulted in the loss of CBs. Nuclear coilin becomes detectable in individual cells when it is concentrated by formation of CBs.

### VRK1 kinase activity is necessary for assembly of Cajal bodies

**Figure 2:**
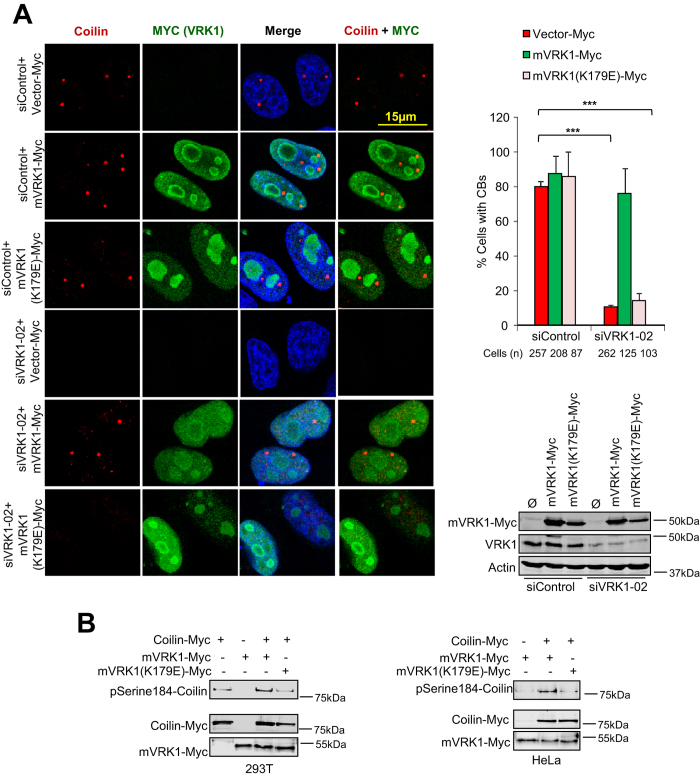
Cajal bodies are rescued by wild-type VRK1 but not by kinase-dead VRK1. **A.** Rescue of defective Cajal Body formation by murine VRK1. HeLa cells were transfected with siVRK1-02 to knock down the endogenous human VRK1, or with siControl. Cells were retransfected with plasmid expressing murine VRK1-myc-DKK, or with the same plasmid containing the K179E substitution that is kinase-dead. After retransfection cells were immunostained with anti-myc polyclonal antibody to identify those cells expressing exogenous VRK1 active or kinase-dead. Cajal Bodies were visualized by staining with anti-Coilin monoclonal antibody Pdelta (SantaCruz). Quantification of the number of Cajal Bodies (graphs) in the rescue experiment were determined using ImageJ software (NIH). Means of the number (percentage) of cells with or without Cajal Bodies and standard deviations are represented in the graph. The number of analysed cells is indicated at the bottom. *(P < 0.05) **(P < 0.005) ***(P < 0.0005). Efficiency of endogenous VRK1 silencing, and expression of murine VRK1 (mVRK1), active or kinase-dead (K179E) were determined by Western blot. **B.** Murine VRK1 that is active, but not its kinase-dead mutant (K179E), phosphorylated coilin in Ser184. Two cell lines, HEK293T (left) and HeLa (right) were transfected with plasmid expressing active murine VRK1, or inactive murine VRK1(K179E), both tagged with a myc epitope. These tagged murine VRK1 were immunoprecipitated and used for an *in vitro* kinase assay with coilin-myc as substrate. Phosphorylation of coilin was detected with a specific antibody for Ser184-P.

Because VRK1 activity is regulated in cell cycle^34^ and also phosphorylates coilin in multiple residues^33^ we tested whether the kinase activity of VRK1 is necessary for the assembly of CBs. For this aim endogenous VRK1 was knocked down, and the rescue of CBs formation was studied by expressing either wild type or kinase-dead (K179E) murine VRK1 (Fig. 2); or alternatively by wild type or kinase-dead (K179E) human VRK1 constructs that are resistant to siRNA (Supplementary Fig. S3). The active murine VRK1, but not its kinase-dead mutant (K179E), also phosphorylates coilin in Ser184 (Fig. 2B).The wild type VRK1 proteins, either murine or the siRNA resistant human protein, were able to rescue formation of CBs (Fig. 2, Supplementary Fig. S3). However, murine and human kinase-dead VRK1 (K179E) proteins were unable to recover CBs (Fig. 2, Supplementary Fig. S3). These results are consistent with the requirement of the VRK1 kinase activity for the assembly of CBs.

### Coilin phosphorylation in Ser184 is associated to the kinase activity of VRK1 in proliferating cells

**Figure 3:**
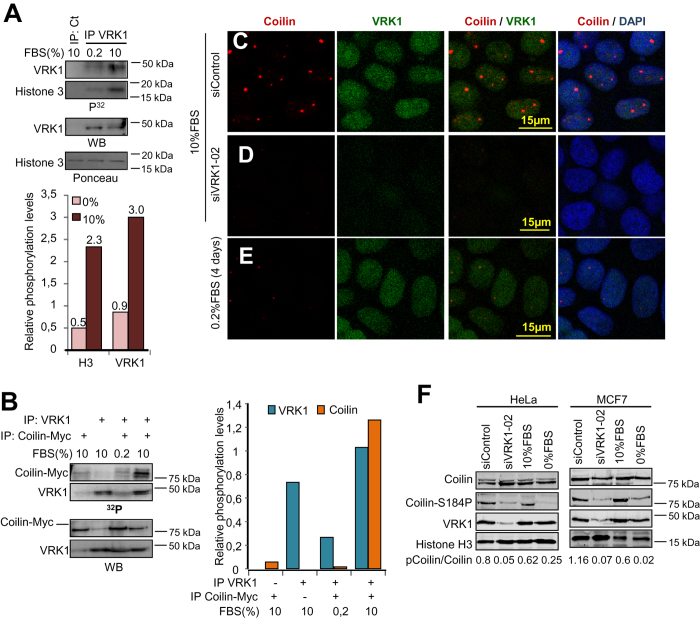
VRK1 activity is necessary for assembly of Cajal bodies. **A.** Kinase activity assay of endogenous VRK1 immunoprecipitated from cells in the absence or presence of serum. The kinase assay was performed using either VRK1 autophosphorylation or histone H3 as substrate. The two top westerns show the radioactive kinase assay. The bottom westerns are loading controls. Quantifications are shown at the bottom. **B**. Endogenous VRK1 was immunoprecipitated from cells in the absence or presence of serum, and its activity determined by *in vitro* kinase assays of autophosphorylation or transphosphorylation using coilin as substrate (upper panel). Quantifications are shown to the right. **C**–**E.** Effect of serum deprivation or VRK1 knock-down on presence of CBs within cells. **F.** Effect of serum deprivation or VRK1 knock-down on coilin phosphorylation in Ser184. Nuclear extracts from HeLa and MCF7 cells were prepared and the indicated proteins detected in western blots. H3 was used as control of the nuclear extract.

The requirement of the VRK1 kinase activity for CBs formation suggested that it might be implicated in CBs assembly during proliferation since both, VRK1^34^,^51^ kinase-activity and CBs formation^4^,^31^, are cell cycle regulated. Coilin is a hyperphosphorylated protein in proliferating cells^4^, and one of eight residues phosphorylated by VRK1 is Ser184^33^. To address if VRK1 affects Ser184 in proliferating cells, MCF7 cells were placed in media with and without serum, endogenous VRK1 was immunoprecipitated and its activity determined in kinase assays that include VRK1 autophosphorylation, and in the phosphorylation of histone H3 (Fig. 3A) and coilin (Fig. 3B) as substrates. Because the activity of VRK1 was lower in the absence of serum than in its presence, we determined if serum deprivation could also result in loss of CBs with respect to control (Fig.3 C–E). Both, serum deprivation and VRK1 knockdown resulted in the loss of CBs (Fig.3 C–E), and of the specific phosphorylation of coilin in Ser184, which were detected in HeLa and MCF7 cells (Fig. 3F). These data indicated that the loss of CBs in arrested cells is associated to the kinase activity of VRK1 (Fig. 3).

### Coilin phosphorylation in Ser184 occurs before assembly of CB

**Figure 4:**
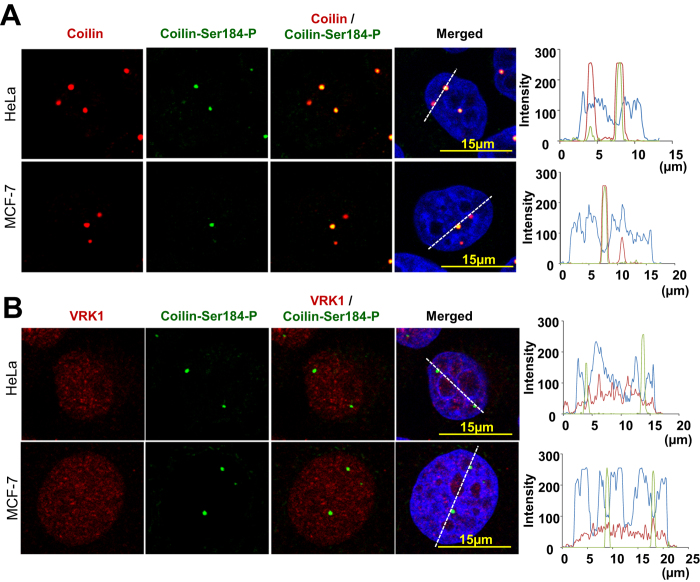
Nuclear localization of VRK1, coilin and coilin-Ser184-P. **A** Colocalization of coilin and coilin phosphorylated in Ser184 in MCF7 and HeLa cells. To the right is shown the profile of the signal to show that some CBs after assembly do not have phosphorylated coilin. **B** Colocalization of VRK1and coilin phosphorylated in Ser184 in MCF7 and HeLa cells. To the right is shown the profile of the signal to show VRK1 is not within CBs with phosphorylated coilin. Field images are shown in Supplementary Fig. S4.

Next we studied whether coilin phosphorylated in Ser184 was present within CBs and whether it co-localizes with VRK1 in two cell lines. Coilin phosphorylated in Ser-184 was detected within CBs, but not all CBs have Ser184-phosphorylated coilin as shown by the fluorescence profile (Fig. 4A). VRK1 was always detected in nucleoplasm and did not co-localize with phosphorylated coilin within CBs (Fig. 4B). This result indicated that after CBs assembly, this specific residue Ser184 may be dephosphorylated, and was not required for CBs maintenance. Nevertheless, coilin within CBs is likely to be regulated by additional phosphorylation in multiple residues that are likely to play different functional roles.

**Figure 5:**
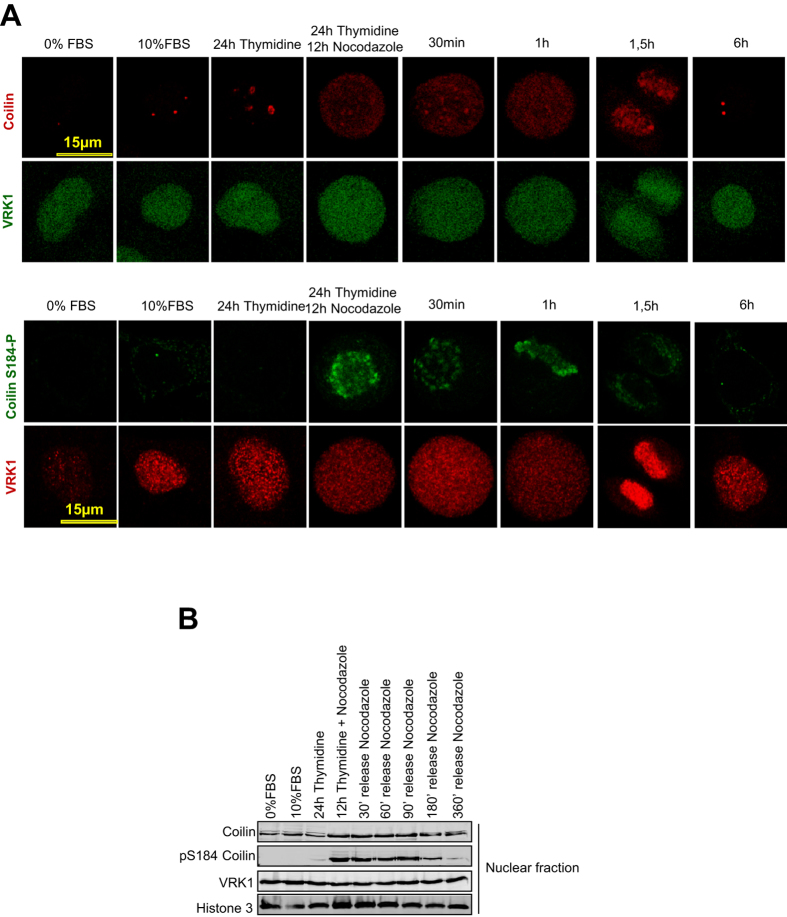
Nuclear localization of VRK1, coilin and coilin-Ser184-P. **A** Colocalization of coilin-Ser184-P, coilin and VRK1 in different phases of cell cycle progression detected by confocal microscopy. Coilin phosphorylated in Ser184 was detected in nuclei of cells arrested with thymidine-nocodazole in G2/M till early G1 after their release, just before CBs assembly. **B** Colocalization of coilin-Ser184-P, coilin and VRK1 in different phases of cell cycle progression detected by immunoblots of the nuclear fraction from cells arrested with thymidine-nocodazole and at different time points after its release. Coilin phosphorylated in Ser184 was detected in time points corresponding to cells arrested in G2/M till early G1, just before CBs assembly.

Next we determined whether the specific phosphorylation of coilin in Ser184 was associated to cell cycle progression (Fig. 5A). For this aim, cells were synchronized with thymidine-nocodazole. The pattern of CBs formation and of coilin phosphorylated in Ser184 is inverted. Coilin Ser184 phosphorylation was mainly detected in mitotic cells, from G2/M to early G1 phase in which there are no CBs, and disappeared when CBs were reassembled, consistent the requirement of this specific residue before assembly, but not for CBs maintenance (Fig. 5A). This phosphorylation of coilin in Ser184 was also studied by immunoblot analysis after thymidine-nocodazole release. Coilin-Ser184 was phosphorylated in G2/M, and after its release it lasted just up to the time in which CBs were reassembled (Fig. 5B). This result is identical to the observation made in immunofluorescence experiments (Fig. 5A, bottom).

### VRK1 depletion reduces coilin levels by facilitating its proteasomal degradation

**Figure 6:**
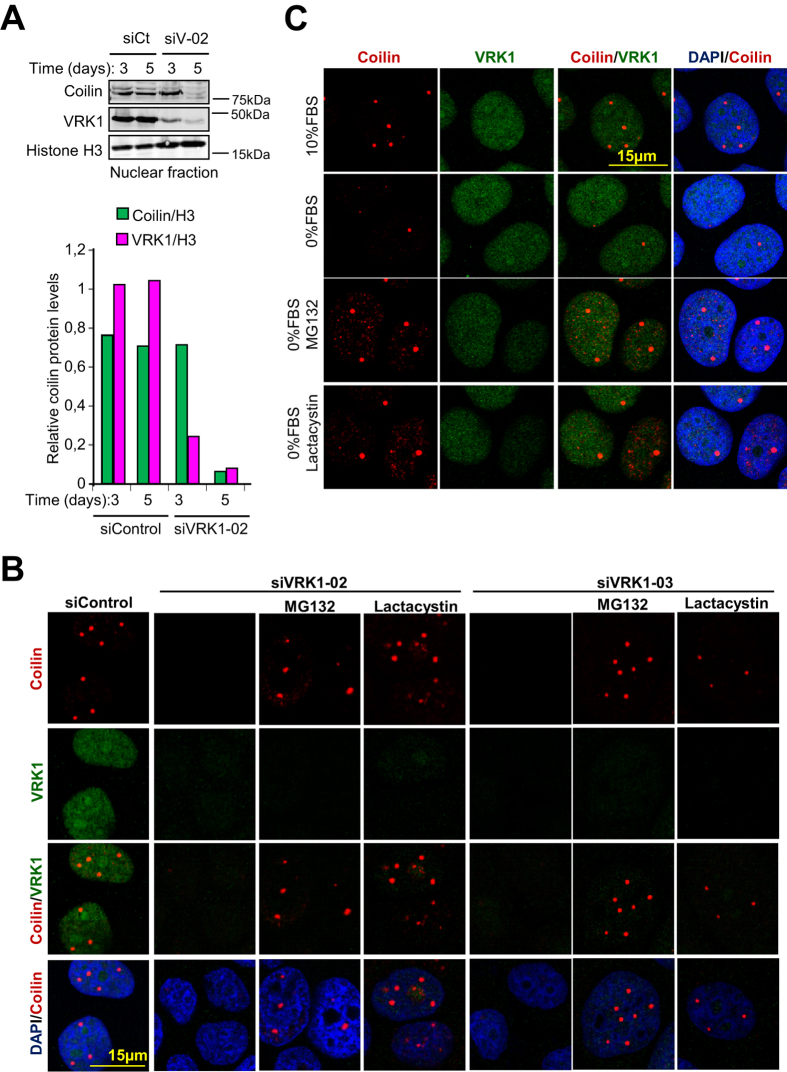
Proteasomal inhibitors protect CBs from VRK1 knock-down and serum deprivation. **A** Levels of coilin in the nuclear fraction in HeLa cells after 5 days of VRK1 depletion. **B** MCF7 cells were transfected with two different siRNA for VRK1: siVRK1-02 or siVRK1-03. Cells were incubated with MG132 (35 μM ) for six hours, or with lactacystin (5 μM ) for ten hours, before cells were lysed at seventy-two hours post knock-down. Cells were processed for immunofluorescence. Colin was detected with Pdelta monoclonal antibody (Santa Cruz), and VRK1 with a polyclonal antibody (Sigma). **C** Serum deprivation causes disintegration of CBs that is protected by proteasomal inhibitors, MG132 or lactacystin.

To elucidate the mechanism by which VRK1 can regulate CBs disassembly we initially determined the temporal effect of VRK1 knock down on the intracellular levels of VRK1 and coilin. The reduction of VRK1 was clearly detectable at three days and preceded the reduction of coilin, which was detected two days later (Fig. 6A). Reduction of endogenous coilin protein levels was similarly achieved with three different siVRK1 in HeLa and MCF7 cells (Supplementary Fig. S5A). This late effect of VRK1 depletion on coilin levels might be either a consequence of downregulation of *COIL* gene expression, or of altering coilin protein stability. Taking into account that VRK1 cooperates with several transcription factors^47^ we first determined whether VRK1 could regulate *COIL* gene expression. Depletion of VRK1 by knock down had no effect on *COIL* RNA expression that was determined by qRT-PCR (Supplementary Fig.S5B). To confirm this effect HeLa cells were transfected with Coilin-myc plasmid expressing coilin from a different promoter, and in this case the knockdown of VRK1 also resulted in a reduction of transfected coilin protein levels (Supplementary Fig.S5C). Based on these observations we concluded that VRK1 affects the stability of coilin protein, but not *COIL* gene expression.

To identify the potential mechanisms by which VRK1 regulates coilin stability we thought that phosphorylated coilin might be more resistant to proteolytic degradation. To test this possibility, VRK1 was knocked down and cells were treated with proteasomal inhibitors, MG132 and lactacystin, and their effect on CBs was determined. Both proteasomal inhibitors were able to prevent the disassembly of CBs as a consequence of the loss of VRK1 (Fig. 6B, Supplementary Fig.4D). This result is also consistent with the protective effect of MG132 and lactacystin on CBs in serum deprived cells (Fig. 6C), in which VRK1 is not active (Fig. 2). Therefore, we concluded that coilin is likely to be degraded in the proteasome, and that this degradation is prevented by VRK1, consistent with the requirement for an active kinase.

### Coilin is an ubiquitinated protein

**Figure 7:**
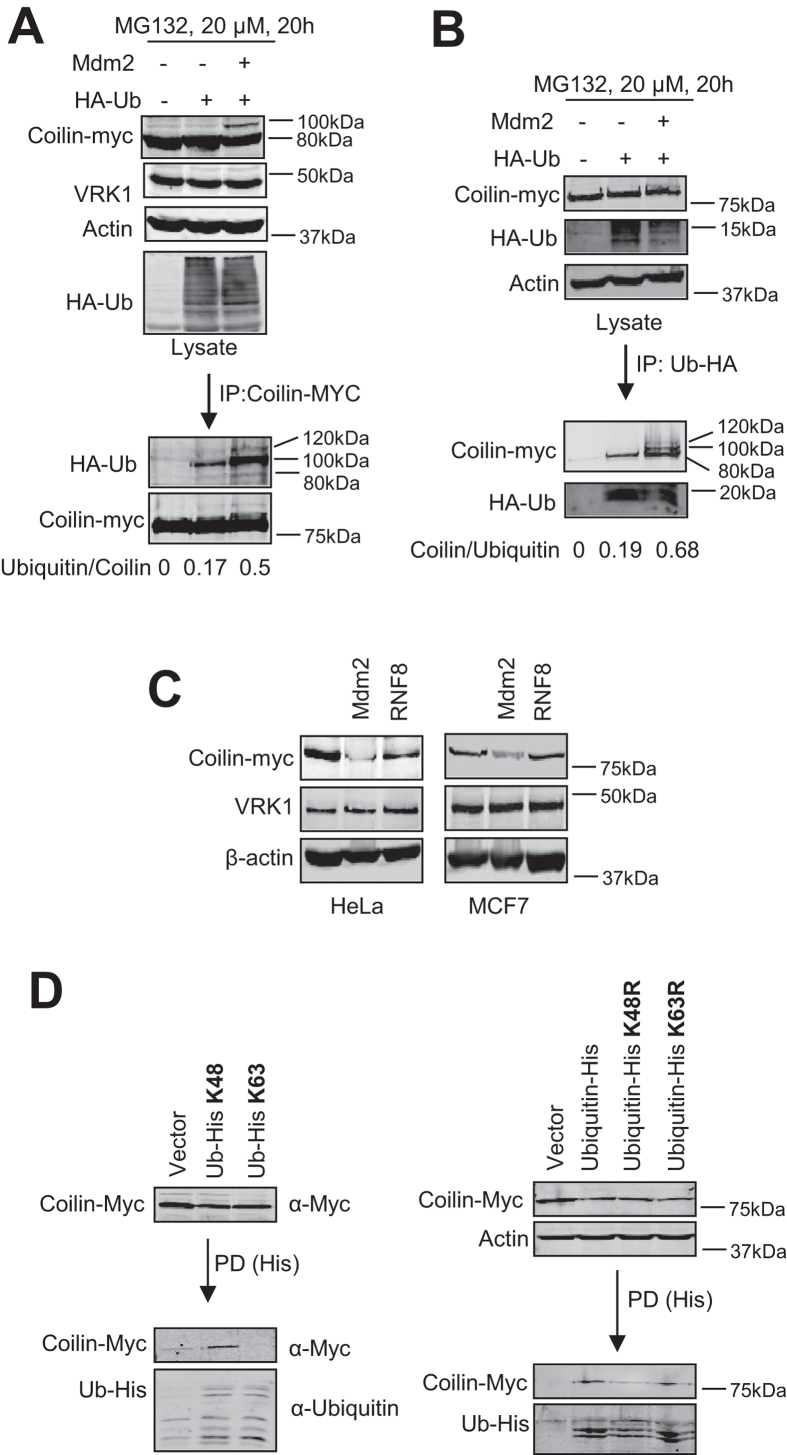
Ubiquitination of coilin. **A** Detection of coilin ubiquitination. MCF7 cells were transfected with plasmids pCMV6-Coilin-myc (5 μg) and pSSK-HA-Ubiquitin (1 μg), with and without pCOC-Mdm2 (4 μg). Coilin was immunoprecipitated and its ubiquitination determined with an anti-HA antibody. **B** Reciprocal experiment to detect coilin ubiquitination. Total ubiquitinated proteins were immunoprecipitated, and the presence of coilin in the immunoprecipitate was determined. **C** Ubiquitin ligases degrading coilin. HeLa and MCF7 cells were transfected with plasmids pCMV6-Coilin-myc (5 μg) and pCOC-Mdm2 (6 μg) or HA-RNF8(6 μg). The levels of the proteins were determined by immunoblot using cell extracts prepared forty-eight hours after transfection. **D** Ubiquitin residue implicated in the ubiquitination of coilin. MCF7 cells were transfected with pCMV6-Coilin-myc and plasmids expressing HA-ubiquitin (wt), or ubiquitin-his constructs in which all lysine residues have been mutated except K48 or K63 (left). Alternatively ubiquitin-His in which only residues K48R or K63R were mutated (right) were used for pulldown. A pulldown of ubiquitin-His was performed using Talon resin, and the presence of coilin determined.

The protective effect of proteasomal inhibitors on CBs suggested that coilin is an ubiquitinated protein. Therefore, we searched for the presence of ubiquitinated coilin. To initially address this point, MCF7 cells were transfected with plasmids expressing coilin-myc, HA-Ubiquitin and mdm2, and some cells were also treated with MG132, a proteasomal inhibitor. In the lysate, ubiquitinated proteins were detected in cells transfected with HA-Ubiquitin (Fig. 7A). Furthermore, an increase in mostly mono-ubiquitinated, and some bi-ubiquitinated, coilin in the coilin immunoprecipitate were also detected in the presence of MG132 (Fig. 7A, bottom). In a reciprocal experiment ubiquitinated proteins were immunoprecipitated, and coilin was also detected in this immunoprecipitate (Fig. 7B).

Cells have multiple ubiquitin ligases^60^. Therefore, we tested ubiquitin ligases that likely candidates to be coilin regulators. Mdm2 and RNF8 were selected because of their implication in processes that are regulated by VRK1 such as p53^40^ and DNA damage responses^51^,^61^. For this aim, HeLa and MCF7 cells were transfected with coilin-myc and plasmids expressing either mdm2 or RNF8 ubiquitin ligases using endogenous ubiquitin. Mdm2, but not RNF8, was able to induce degradation of coilin (Fig. 7C) without affecting VRK1 (Fig.7C). Next we tested whether mdm2 or RNF8 were able to form a detectable complex with coilin. For this aim cells were transfected with tagged Mdm2 or RNF8 in combination with coilin. Mdm2 (Supplementary Fig.S6A), but not RNF8 (Supplementary Fig.S6B), was able to interact with coilin in reciprocal immunoprecipitations. To determine which ubiquitin residue is used in the initial ubiquitination of coilin, plasmids expressing ubiquitin mutated in all lysine residues, except K48 or K63 were used. Coilin ubiquitination was only detected when ubiquitin reacting only by K48 was used, but not with ubiquitin reacting by K63 (Fig. 7D, left). The use of this residue was confirmed using ubiquitin plasmids, in which all lysines were available for reaction, except the mutated K48R or K63R. In this case only the wild type or K63R mutant ubiquitinated coilin, since in both K48 is available for ubiquitination. This was confirmed by using the K48R mutant that did not ubiquitinate coilin (Fig. 7D, right). These data indicated that ubiquitin K48 is the residue used in the ubiquitination of coilin.

### Blocking nuclear export prevents degradation of ubiquitinated coilin

**Figure 8:**
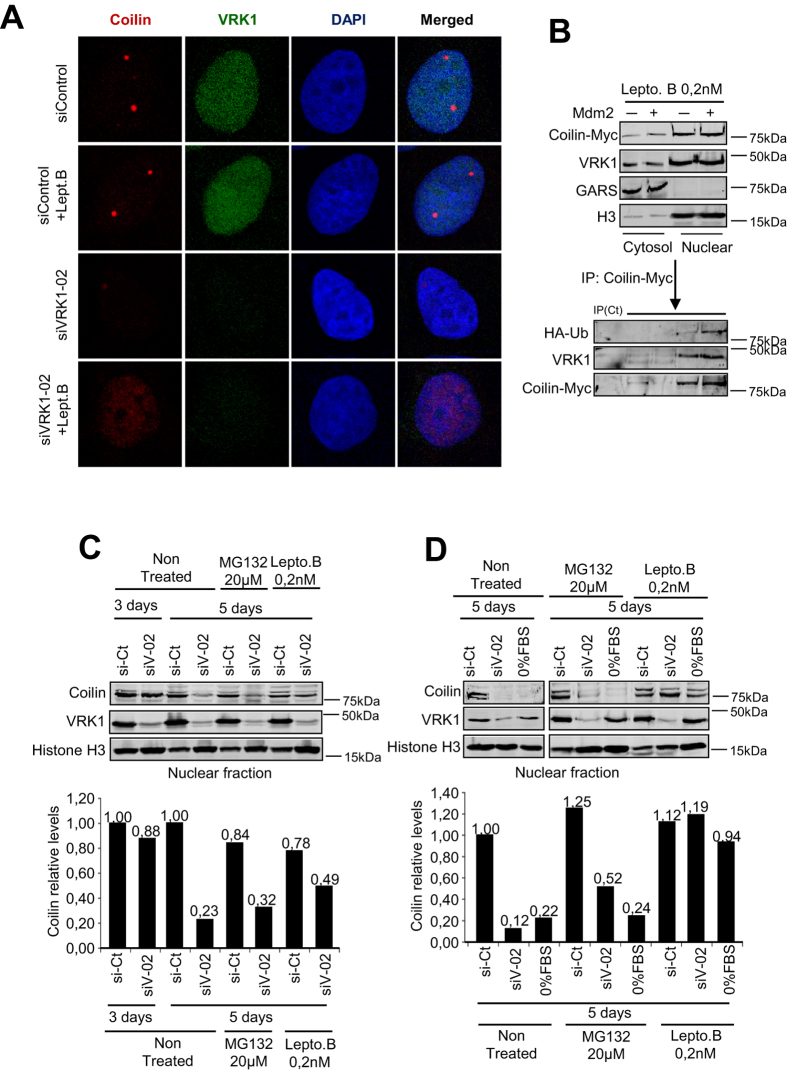
Nuclear export is necessary for degradation of ubiquitinated-coilin. **A** MCF7 cells, either control or in which VRK1 was knocked-down, were treated with leptomycin B and its effect on the presence of coilin in nuclei determined by immunofluorescence confocal microscopy. **B** Accumulation of ubiquitinated coilin in nuclei in cells treated with leptomycin B. **C** Effect of MG132 or leptomycin B on the level of nuclear coilin in cells in which VRK1 was knocked-down. **D** Effect of leptomycin B on coilin nuclear levels after serum deprivation or after VRK1 knock-down.

Proteasomal degradation is a complex process that takes place either in cytosol or nuclei. To determine where coilin was being degraded, endogenous VRK1 was knocked-down and cells were treated with leptomycin B at 0.2 nM to inhibit the nuclear export of proteins. This treatment with leptomycin B resulted in the accumulation of coilin within nuclei, but not in CBs (Fig. 8A). Thus we determined whether there was an accumulation of ubiquitinated coilin within nuclei of cells that were treated with leptomycin B. For this aim cells were transfected with ubiquitin and mdm2 and nuclear export inhibited with leptomycin B, under these conditions ubiquitinated coilin accumulated in the nuclear fraction (Fig. 8B). Also the nuclear accumulation of endogenous coilin was observed in cells treated with leptomycin B (Fig. 8C). A similar accumulation of coilin within nuclei was detected in cells treated with leptomycin, but not by MG132, following VRK1 knockdown or serum deprivation (Fig. 8D). Therefore we concluded that coilin is ubiquitinated in the nucleus, but its degradation in the proteasome requires its exit to the cytosol.

## Discussion

Coilin is a hyperphosphorylated protein in proliferating cells^4^. In this work we have identified the mechanisms by which VRK1 regulates the assembly and stability of CBs by phosphorylation of coilin. Coilin forms a molecular complex of 160 kDa (Fig. 1B), which is not affected by VRK1 depletion, and is likely to represent the basic unit from which CBs are assembled in a VRK1-dependent manner. In this context kinases which are regulated in cell cycle are likely candidates. This process is dependent on the kinase activity of VRK1 since kinase-dead VRK1 is unable to rescue the disintegration of CBs due to VRK1 knockdown. The regulation of VRK1 activity in cell cycle and the parallel phosphorylation of coilin suggest a role for VRK1 in proliferation- dependent assembly of CBs and are consistent with a nuclear phosphorylation of coilin before CBs assembly. VRK1 is regulated in cell cycle and its expression and activity increases in the G1/S phase^34^, and predicts that effects of serum deprivation are likely to be similar to VRK1 depletion. In this context, VRK1 specific phosphorylation of coilin in Ser184^33^ is associated to cell proliferation in the presence of serum, and to the regulation of VRK1 activity. However, other kinases have been identified within assembled CBs, such as CDK2/cyclin E^32^ and which are likely to participate in interactions and regulation of processes taking place within CBs. Since coilin can be phosphorylated in multiple residues^4^,^28^, the pattern of coilin phosphorylation within CBs is likely to determine the role of coilin in different biological processes taking place within CBs as well as its stability. No clear role has been attributed to most of the residues phosphorylated in coilin. Phosphorylation of Ser184, one of the residues targeted by VRK1^33^, has been associated to cell cycle regulation and occurs mainly in mitosis and precedes assembly of CBs (this report). Coilin Ser489 has been implicated in the interaction of coilin with RNA^16^, and can also contribute to CBs disruption^30^. Phosphorylation of coilin in its C-terminal region also modulates its interactions with SMN or SmB’^25^. VRK1 by phosphorylating coilin contributes to its stability and thus to nucleation of CBs bodies on coilin. Additionally phosphorylated coilin residues can contribute to the retention of other proteins, such as IntS4^62^ or Fam118B^63^ whose loss also results in CBs disintegration. Furthermore, these additional phosphorylations might be an active mechanism for retaining proteins as part of CBs, such as SMN1 or gemins among others^17^,^22^. CBs are associated with the splicing machinery, and perhaps sequestration of splicing machinery in CBs during cell cycle progression might be cellular mechanisms by which transcription and translation are switched to a replication mode, with its subsequent reorganization of chromatin structure, a role that is also dependent on VRK1 activity^36^.

Loss of coilin in cells arrested by serum deprivation is a likely consequence of its degradation. Coilin is able to interact with mdm2/hdm2 ubiquitin ligase that ubiquitinates coilin resulting in its proteasomal degradation as demonstrated by its sensitivity to inhibitors, such as MG132 and lactacystin. Since VRK1 and mdm2 do not appear to accumulate in CBs, the effect of VRK1 might be due to its effect on nuclear coilin not incorporated to CBs, or alternatively released from them. The consequence of nuclear export and degradation of coilin displaces the equilibrium of CBs to their disassembly, since CB formation is a consequence of coilin levels. VRK1 knockdown, as well as serum deprivation, facilitated coilin ubiquitination. The ubiquitin residue used in the reaction partly determines the fate of the ubiquitinated protein^60^. Ubiquitination by mdm2 is mostly associated to nuclear export^64^ and ubiquitination by the K48 residue is mainly associated with degradation reaction^65^. Both affect coilin and these may partly account for its degradation in the cytoplasm. Blocking nuclear export with leptomycin B resulted in the retention and detection of ubiquitinated coilin dispersed within nuclei (Fig. 8). The use of leptomycin B at a concentration one-hundred and fifty fold higher resulted in coilin accumulation within nucleolus^66^. There are more than six hundred ubiquitin ligases^60^, indicating that ubiquitination is a selective and highly regulated process, with specificity is not yet fully understood. This separation in different compartments allows ubiquitination to have alternative functional roles depending both on number and branching of ubiquitinated residues, as well as the resulting conformational changes that can regulate its interactions with other proteins^67^.

The regulation of CBs assembly in proliferating cells can have functional consequences for this organelle or its components. One of them is making accessible, or sequestering, different proteins depending on the phase of cell cycle. These proteins are likely to be free or in a different organization during interphase, and be sequestered and concentrated at the time of mitosis; in that way cells can switch from a transcription to a replication mode^17^. Among protein candidates to be regulated by sequestration in CBs are components of splicing and RNA processing machineries, which might have a specific and different roles in cell cycle or non-dividing cells. For example, SMN is a CB component that in arrested cells is free in nuclei and cytosol, but that is partly sequestered in CBs during cell proliferation^68^. Furthermore, CBs can also concentrate factors implicated in telomere maintenance^17^, and thus be different depending on the proliferation situation.

**Figure 9:**
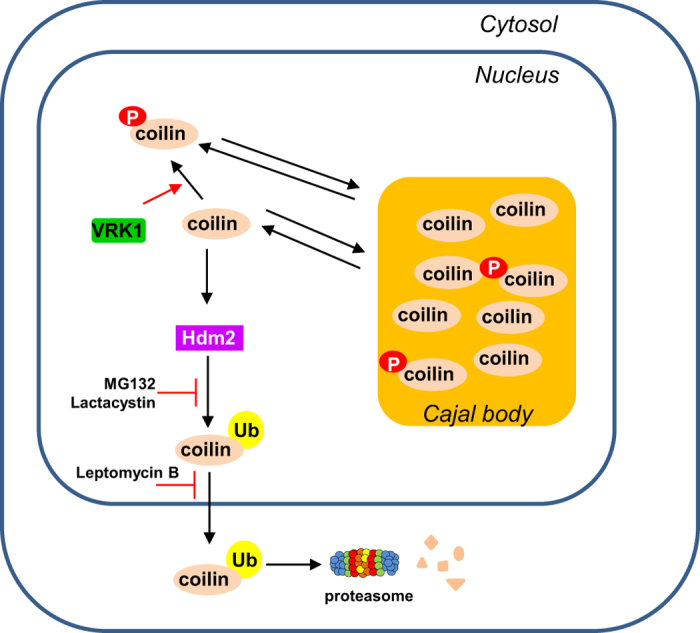
Model of Cajal Body regulation by VRK1.

We propose a model, in which VRK1 mainly controls the stability of nuclear coilin, which if stabilized, can lead to its aggregation and assembly of CBs (Fig. 9). In case that VRK1 is inactive, it will permit a drop in nuclear coilin and facilitate disassembly of CB and release of its components. Thus, it will be necessary to study the fate of other CB components such as SMN1 in the absence of VRK1 since they might provide clues to its role in the pathogenesis of a complex neurological phenotype associated to VRK1 mutations and that cause a pontocerebellar hypoplasia accompanied by muscular atrophy and ataxia^69^,^70^,^71^,^72^. VRK1 might be a common nexus among neurological phenotypes associated to the motor neuron, and opens up an important research area.

## Materials and Methods

### Plasmids

Plasmids expressing active and kinase-dead human VRK1, pCEFL-HA-VRK1 and pCEFL-HA-VRK1(K179E) have been described previously^38^,^41^,^42^,^48^,^51^. Plasmid pCEFL-HA-VRK1(R391/R393/V394) resistant to si-VRK1-01 has been described^33^. Murine VRK1 was expressed from plasmid pCMV6-mVrk1-myc-DKK (OriGene, Rockville, MD). A kinase-dead construct of the murine VRK1(K179E) was generated with the Quick-Mutagenesis system (Stratagene, San Diego, CA) using the following primers (forward: 5′ -GTGCACGGGGACATCGAGGCCTCCAACCTGCTCCT -3′; reverse: 5′ -AGGAGCAGGTTGGAGGCCTCGATGTCCCCGTGCAC -3′. Coilin was expressed from plasmid pCMV6-coilin-myc-DKK (Origene, Rockville, MD); mdm2 from pCOC-Mdm2-X2 (M. Oren, Rehovot, Israel)^43^; ubiquitin from pSSK-HA-Ubiquitin and pHis-Ubiquitin (D. Lane, Dundee, UK) and HA-RNF8 (T. Thomsom, IBMB-CSIC, Barcelona, Spain)^73^. Ubiquitin was expressed from different constructs: pcDNA3- 6xHis-Ubiquitin(wt), pcDNA3-6xHis-Ubiquitin K48R, pcDNA3-6xHis-Ubiquitin K63R, (J. Lozano, University of Malaga, Spain); Ubiquitin-HA-His K63 (all Lys mutated except K63) and Ubiquitin-HA-His K48 (all Lys mutated except K48)^74^.

### Cell culture and transfection

The following cell lines were used in this work: SH-SY5Y, HEK293T, HeLa, MCF7, WS1, HCT116 and A549 all obtained from the ATCC-LGC (Teddington, UK). Cells were authenticated and grown as recommended by supplier.

### VRK1 knock-down

VRK1 knockdown was performed using three different siRNA specific for VRK1 (Accession number NM_003384): siVRK1-01 (siV-01), siVRK1-02 (siV-02), siVRK1-03 (siV-03) and siVRK1-09 (siV-09) (all from Dharmacon RNA Technologies- GE Healthcare). The sequences targeted by these VRK1 siRNA oligonucleotides were siVRK1-01: GAAAGAGAGTCCAGAAGTA; siVRK1-02: CAAGGAACCTGGTGTTGAA; si-VRK1-03: GGAAUGGAAAGUAGGAUUA; and siVRK1-09: AGGUGUACUUGGUAGAUUA. As negative control the “ON-TARGETplus siCONTROL Non-targeting siRNA” (siCt) (Dharmacon) was used. The efficiency of RNAi transfection was determined with “siGLO RISC-free siRNA” (DHARMACON) labelled with a red fluorochrome. All of them have been previously used^34^,^48^,^51^.

Briefly, cells were transfected with the indicated siRNA at a concentration of 20 nM using Lipofectamine 2000 Reagent” (Invitrogen) according to manufacturer instructions. After transfection cells were processed for specific experiments at the times indicated in them. For rescue experiments, cells were transfected with the indicated siRNA using Lipofectamine 2000 Reagent, and 36 hours later, cells were retransfected with plasmids using JetPI reagent (Poly Plus, Ilkirch, France) according to manufacturer instructions. Targeted protein and plasmid expression were analysed thirty-six hours after the second transfection^51^.

### High-performance liquid chromatography (HPLC)

Cells were lysed 72 hours post-transfection in Lysis Buffer (20 mM Tris-HCl pH 7.4, 137 mM NaCl, 2 mM EDTA, 25 mM β-glycerophosphate, 10% v/v glycerol, 1% Triton-X-100, protease and phosphatase inhibitors). Insoluble material was eliminated by centrifugation at 13200 rpm 20 minutes in an Eppendorf 5415R centrifuge. Soluble material was quantified and 3 mg of protein were fractionated by HPLC in a Superose 12 10/300 GL column (Pharmacia-GE Healthcare). Fractions were eluted in Elution Buffer (50 mM Tris HCl, pH 7.5, 1 mM EDTA, 100 mM KCl, sodium azide 0.025%), previously filtered (0.22 μm) and degassed, at a flow rate of 0.4 ml/min and monitored at 280 nm. Fractions of 0.2 ml were collected, precipitated and resolved on polyacrylamide gels and the following molecular weight markers (BioRad) were used: bovine thyroglobulin (670 kDa), bovine γ-globulin (158 kDa), chicken ovalbumin (44 kDa), horse myoglobin (17 kDa) and vitamin B12 (1.35 kDa).

### Nuclei isolation

HeLa cells were plated on 100 mm dishes and 48 hours later the cells were washed and harvested by centrifugation at 600 g for 5 minutes. Pelleted cells were resuspended in 10 volumes of RSB buffer (0.01 M NaCl, 1.5 mM MgCl_2_, 0.01 M Tris HCl pH 7.4) and incubated on ice 10 minutes. At this point a small aliquot was collected to examine the cells in a microscope (if the cells have “ballooned” swollen cytoplasm, the preparation is ready for the next step). Then, cells were introduced into a glass Dounce-type homogenizer and were subjected to 10-12 quick upward strokes. Once again, cells were examined in a microscope (now >90% were free nuclei). Suspension was centrifuged at 1000 g for 3 minutes (4 °C) to pellet the nuclei. The pellet was washed three times with RSB buffer.

### Cajal Body Isolation

All the procedures were performed on ice. S1 solution (0.25 M sucrose, 10 mM MgCl_2_) was added to HeLa nuclei to reach a final volume of 30 mL. 15 mL of Solution 2 (0.35 M sucrose, 0.5 mM MgCl_2_) were added in two 50 mL Falcon tubes and resuspended nuclei were overlayed onto Solution 2 (15 mL/Falcon). These tubes were centrifuged at 1430 g for 5 minutes (4 °C) and the supernatant was decanted carefully. The pellets were resuspended in 30 mL of Solution 2 and each solution was divided in three 15 mL Falcon tubes to improve sonication (3 × 6 seconds). In this step, the solution was examined in the microscope (nuclei should be lysed and the nucleoli should be visible). The aliquots were pooled together in a 50 mL Falcon tube and 0.42x volume of 2.55 M sucrose was added (final concentration 1 M). The mixture was divided into two portions, in 50 mL Falcon tubes (approximately 20 mL each portion), and were centrifuged at 3000 g for 10 minutes (4 °C). The supernatant (contains the nucleoplasm with Cajal bodies) was collected with pipette. After that, 0.82x volume of SP1 Buffer (1 M sucrose, 34.2% Percoll, 22.2 mM Tris-HCl pH 7.4, 1.11 mM MgCl_2_) and 0.05x volume of 20% Triton were added. This mixture was divided into pre-cooled SW41 ultracentrifuge tubes and they were centrifuged at 37000 rpm for 2 hours (4 °C). The pellet was transferred into a 15 mL Flacon tube and 600 units of DNase1 were added for 1 hour incubation at RT in a rotating wheel. After the incubation with DNase1, 0.05x volume of heparin (10 mg/mL) was added to the mixture. At this point, 1x volume of SP2 (20% Percoll, 10 mM Tris-HCl pH 7.4, 1% Triton X100, 0.5 mg/ml heparin) was added and incubated in a rotating wheel for 1 minute. The mixture was loaded in tubes for SW55 rotor, and centrifuged at 45000 rpm for 1 hour. After centrifugation, three bands were visible in the tube and, the middle band containing the Cajal Bodies was collected. Now, 10x volume of HT Buffer (10 mM Tris-HCl pH 7.4, 1% Triton X100, 0.5 mg/ml heparin) was added and incubated in a rotating wheel. This mixture was divided in Eppendorf tubes and centrifuged at 14000 rpm for 15 minutes. The supernatants were decanted and the pellets were resuspended in 500 μl of HT Buffer. The pellets were pooled together in one tube and centrifuged at 14000 rpm for 15 minutes. Again the supernatant was eliminated and the pellet was resuspended in 500 μl of S3 solution (0.5 M sucrose, 25 mM Tris-HCl pH 9.0). This mixture was centrifuged at 8000 rpm for 5 minutes, and the supernatant was centrifuged another time in the same conditions and transferred to a new tube. The supernatant was diluted with 10x volume of S3 solution and centrifuged at 14000 rpm for 15 minutes. Once more, the supernatant was eliminated and the pellet was resuspended in S3 solution and centrifuged at 14000 rpm for 15 minutes. Finally, the supernatant was eliminated; leaving 20 μl in the tube in which the pellet was resuspended obtaining the fraction enriched in Cajal Bodies.

### Antibodies

The following antibodies, type and dilution were used. VRK1 polyclonals VE and VC (1:1000 for immunoblots)^33^,^41^,^48^,^51^,^75^,^76^; mAb 1B5 or 1F6 (1:1000 immunoblots; 1:200 immunofluorescence)^33^,^41^,^48^,^51^,^75^,^76^. VRK1 (Sigma, HPA000660, 1:200 for immunofluorescence). Flag epitope (mAb M5 at 1:1000 or polyclonal F7425 at 1:1000, both from Sigma); HA epitope (Santa Cruz: mAb sc-7392 at 1:1000 for immunoblots; Sigma H6908 at 1:200 for immunofluorescence, and at 1:1000 for immunoblots). β-actin (mAb C-15 at 1:5000 for immunoblots). Histone H3 (Cell Signaling 9715 at 1:1000 for immunoblots). Mdm2 (Pierce, Ab PA5-27237 at 1:1000 for immunoblots). GARS (Santa Cruz, sc-98614, at 1:200 for immunoblots and 1:50 for immunofluorescence). Coilin (Santa Cruz, sc-56298, Pdelta; sc-32860, H300). Coilin-Ser184P was affinity purified with a Ser184P phospho-peptide (APS, Shefford, UK, at 1:1000 for immunoblots and 1:200 for immunofluorescence). RB (Santa Cruz, sc-50; 1:1000, phospho RB (Ser807/811)(Cell Signaling, 1: 1000 for immunoblots) , cyclinD1 (Santa Cruz, sc-450 at 1:500 for immunoblots). The secondary antibodies used for immunoblots using chemiluminiscence: α-mouse IgG, horseradish peroxidase-linked species-specific whole antibody (Amersham-GE Healthcare) or α-rabbit IgG (whole molecule) peroxidase conjugate (SIGMA-ALDRICH) and were developed with an ECL kit (Amersham-GE Healthcare). The secondary antibodies used for immunofluorescence in a Li-Cor Odyssey system (Thermo-Fisher) were goat α-Mouse IgG, DyLight^TM^ 680 and/or goat α-Rabbit Ig-G, DyLight^TM^ 800 (Thermo scientific).

### Confocal immunofluorescence microscopy

Cells were plated on 35 mm dishes with coverslips and transfected 24 hours later with expression plasmids or siRNA. Two or three days after, respectively, the slides were collected and the cells were fixed with 3% paraformaldehyde for 30 min at room temperature. Then the cells were treated with a solution of glycine 200 mM for 15 min, and then permeabilized with PBS 0,2% Triton X100 for 30 min. The cells were blocked with 1% BSA in PBS for 1 hour. Proteins of interest were detected by immunostaining with specific antibodies that were incubated overnight or for 1 hour depending on commercial instructions. The secondary antibodies were incubated together (goat anti-mouse Cy3 or Cy2 and goat anti-rabbit Cy3 or Cy2 from Jackson ImmunoResearch) for 1 hour at room temperature. Finally cells were stained with DAPI (4´, 6´-diamidino-2-phenylindole) (Sigma) 1:1000 in PBS for 10 min at room temperature, the cells were washed with PBS and slides were mounted with Mowiol (Calbiochem-Merck, Darmstadt Germany), and analysed with a LEICA SP5 DMI-6000B confocal microscope. The lasers used were: Argon (488 nm), DPSS (561 nm) and UV Diode (405 nm). Images were captured with a 63.0x lens zoomed in 1.5 − 3×with a 1024 × 1024 frame and 600 Hz scanning speed. Microscope scanner settings were maintained constant for image capture for all samples. Images were analysed with ImageJ (NIH, http://rsb.info.nih.gov/ij) and LEICA LAS AF (Leica) software.

### Rescue experiments

Rescue of defective Cajal Body formation was determined using VRK1 constructs resistant to siRNA. HeLa cells were transfected with siVRK1-01 to knock down endogenous human VRK1 or with siVRK1-02 (in rescue experiments with murine VRK1), or with siControl, thirty-six hours later cells were retransfected with 3 μg of plasmid HA-VRK1(R391/R393/V394), with 3 silent substitutions that render the cDNA insensitive to si-VRK1-01^51^ or with murine VRK1. Rescue was also done with the same plasmids containing the K179E substitution that makes murine and human VRK1 kinase inactive^48^.Thirty-six hours after retransfection cells were immunostained with anti-HA or anti-myc polyclonal antibodies to identify cells expressing exogenous VRK1. Cajal Bodies were visualized by staining with Coilin monoclonal antibody Pdelta (SantaCruz). Efficiency of endogenous VRK1 silencing, and expression of siRNA-resistant VRK1 was determined by Western blot. The quantification of the number of Cajal Bodies in rescue experiments was performed using ImageJ software (NIH).

### Immunoprecipitation and GST pull-down assays

Cells were plated on 100 mm dishes and harvested 48 hours after transfection. The cells were lysed with lysis buffer (1%Triton X100, 200 mM NaCl, 5 mM EDTA, 50 mM Tris pH 8.00, 1 mM NaF, 1 mM sodium orthovanadate and protease inhibitors: 10 μg/mL leupeptin, 10 μg/mL aprotinin, 1 mM PMSF), and after incubation on ice for 20 minutes, the extracts were centrifuged at 13200 rpm for 20 minutes at 4 °C. 40 μg of total protein lysate was analysed in SDS-polyacrylamide gel and protein expression checked by Western blot. For immunoprecipitation, 1-2 mg of total lysate was incubated with the specific antibodies for 6 to 8 hours, followed by incubation with Gamma-Bind G Plus Sepharose beads (Amersham) overnight at 4 °C^38^,^42^. Beads were softly washed with lysis buffer 4-5 times, then resuspended in Laemmli Buffer, heated at 100 °C and analysed by SDS-Page. For GST pull-down assays, 1 mg of total cell extract was incubated with Glutathione-Sepharose beads (Amersham) for 6 hours. Beads were washed 4-5 times with lysis buffer, resuspended in Laemmli Buffer and heated at 100 °C. The proteins were analysed by SDS-PAGE.

### Kinase assay

Kinase assays were performed as previously reported^33^,^77^. Briefly, MCF-7 cells were transfected with pCMV6-Coilin-Myc-Flag or with an empty vector. 24 hours after transfection medium without FBS was added and 48 hours later the cells were harvested and proteins were immunoprecipitated. Kinase assays were performed in a final volume of 53 μl containing kinase buffer (20 mM Tris–HCl, pH 7.5, 5 mM MgCl2, 0.5 mM dithiothreitol (DTT), 150 mM KCl), 5 μM ATP and 5μCi of [32P]ATP[γP] (PerkinElmer). In each reaction 1 mg of immunoprecipitated protein and 1 μg of recombinant Histone 3 were used 77. The reactions were incubated at 30 °C for 30 minutes in a Thermomixer (Eppendorf). After the incubation, samples were boiled in Laemmli buffer at 100 °C for 5 minutes. The phosphorylated proteins were analyzed by electrophoresis in 10% or 12,5% SDS-polyacrylamide gels. Proteins were transferred to Immobilon-P membranes (Millipore) and the incorporated radioactivity was measured.

### Immunoblots

Total protein extracts were quantified using a BIORAD Protein assay kit (Biorad, Hercules, CA). Proteins were fractionated in an SDS-polyacrylamide gel and transferred to a PVDF Immobilon-P or Immobilon-FL membranes (Millipore). The membrane was blocked with TBS-T buffer (25 mM Tris, 50 mM NaCl, 2.5 mM KCl, 0.1% Tween-20) and 5% defatted-milk. Afterwards filters were rinsed with TBS-T buffer and the specific primary antibody (indicated in individual experiments) added and incubated for 90 minutes at room temperature. The filter was rinsed and incubated with the corresponding conjugated secondary antibody. Signals were detected by chemiluminescence (ECL, Amersham) or fluorescence (Li-Cor Odyssey).

### qRT-PCR

RNA was analysed and quantified using a Bioanalyzer 2100 nano-lab chip from Agilent Technologies (Germany). 100 ng of total RNA were used in a one-step reverse transcription real-time PCR amplification reaction using the Quantitec SYBR Green RT-PCR kit from Qiagen in an iCycler (BioRad, Hercules, CA). The reaction was analysed with iCycler software (BioRad). The primers used for coilin amplification were: (forward: 5′-TTACCCCCAGCAAGGGCAAGACCTC-3′; reverse: 5′-GGCCTACTGTCTTTAAGAAACCCGCAGCA-3). GAPDH amplification was used as internal control (GAPDH-F: 5′-GGTCTTACTCCTTGGAGGCCATGT-3′; GAPDH-R: 5′-ACCTAACTACATGGTTTACATGTT-3′). VRK1 was amplified with primers (F: 5′-GAGGCCATACAGACCCGTTC-3′; R: 5′-TCCACCTCGCAAGACTCACA-3′)

### Drugs and treatments

Proteasome inhibitors MG132 and Lactacystine were used at 20 μM for 20 hours or at 5 μM for 10 hours, respectively. The nuclear export inhibitor Leptomycin B was used at 0.2 nM for 24 hours.

## Additional Information

**How to cite this article**: Cantarero, L. *et al.* VRK1 regulates Cajal body dynamics and protects coilin from proteasomal degradation in cell cycle. *Sci. Rep.* **5**, 10543; doi: 10.1038/srep10543 (2015).

## Supplementary Material

Supplementary Information
