# Supplementary material for: VRK1 regulates Cajal body dynamics and protects coilin from proteasomal degradation in cell cycle
Source: Sci Rep. 2015 Jun 12;5:10543. doi: 10.1038/srep10543 (PMC4464288; doi:10.1038/srep10543)
Supplement: Supplementary Information [file srep10543-s1.pdf]

Supplementary Information

VRK1 regulates Cajal bodies dynamics and protects coilin from proteasomal degradation in cell cycle

Lara Cantarero [1,2], Marta Sanz-García [1], Hadar Vinograd-Byk [3], Paul Renbaum [3], Ephrat Levy-Lahad [3] and Pedro A. Lazo [1,2,*]

[1] *Experimental Therapeutics and Translational Oncology Program, Instituto de Biología Molecular y Celular del Cáncer, Consejo Superior de Investigaciones Científicas (CSIC), Universidad de Salamanca, Salamanca, Spain*

[2] *Instituto de Investigación Biomédica de Salamanca (IBSAL), Hospital Universitario de Salamanca, Salamanca, Spain*

[3] *Medical Genetics Institute, Shaare-Zedek Medical Center, Hebrew University of Jerusalem Medical School, Jerusalem, Israel*

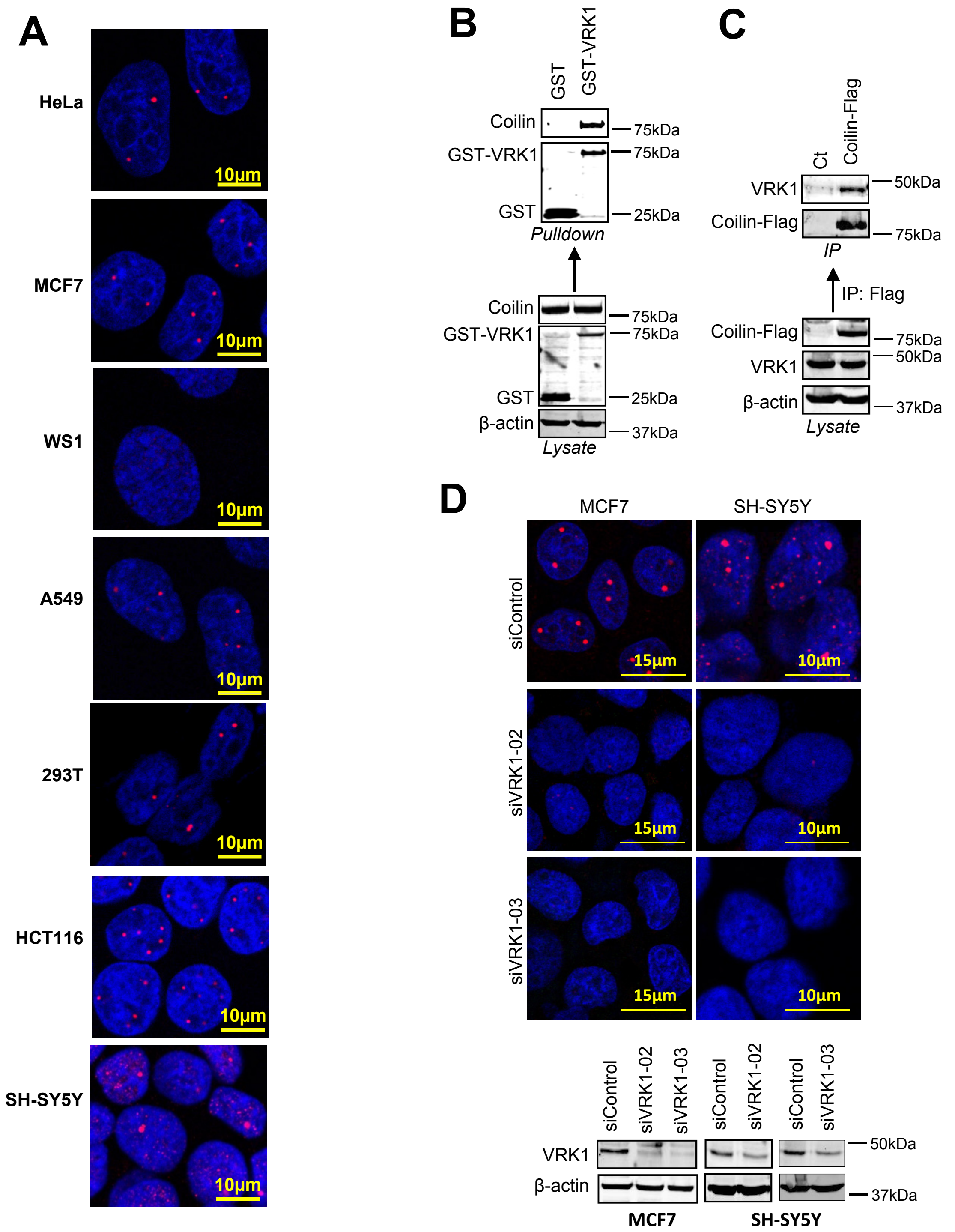

Supplementary Figure S1. Detection of Cajal bodies and effect of VRK1 knockdown. A. Detection of Cajal bodies in a panel of seven cell lines. Coilin was detected with a monoclonal antibody (Pdelta from Santa Cruz) **B**. **Transfected VRK1 interaction with endogenous coilin.** HEK-293T cells were transfected with pCEFL-GST-VRK1 or pCEFL-GST plasmids. Protein expression was determined in the lysates used for pull down.(bottom). Proteins present in the pulldown are shown at the top. **C. Transfected coilin interaction with endogenous VRK1**. HEK293T cells were transfected with plasmid p-Coil-Flag. Protein expression in lysates is at the bottom. Proteins present in the anti-Flag immunoprecipitate are shown at the top **D. Effect of VRK1 knockdown on CBs in MCF-7 and SH-SY5Y cells.** Knockdown was performed with two different siRNAs specific for VRK1.Top: Immunofluorescence confocal microscopy of Cajal bodies detected with an anti-coilin antibody. Bottom: Detection of the level of VRK1 protein in immunoblots detected with an anti-VRK1 (1F6) mAb. Field image and quantitative data for MCF7 cells are shown in Supplementary Fig. 3.

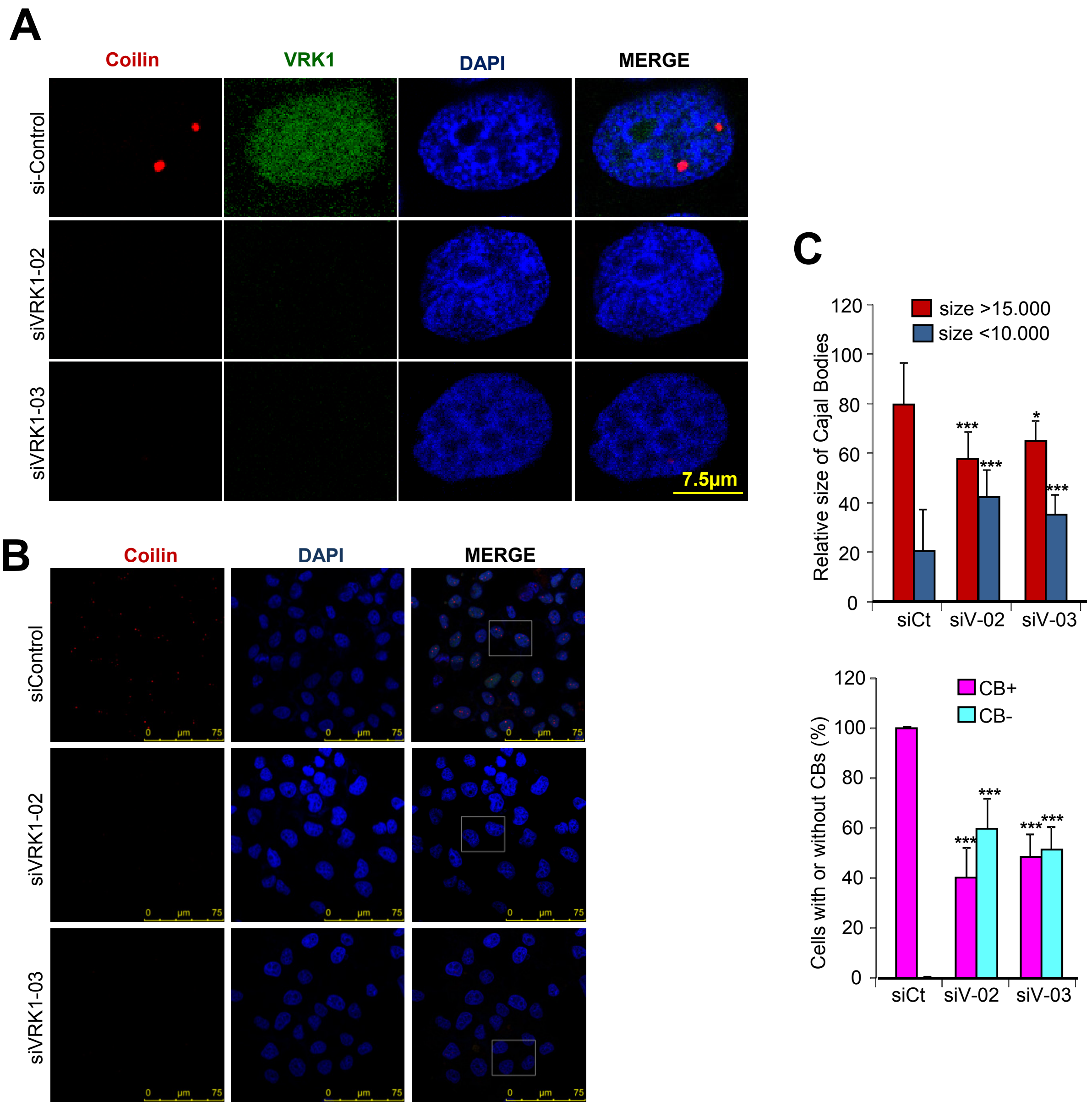

Supplementary Figure S2. VRK1 knockdown results in loss of Cajal bodies in MCF7 cells. A. VRK1 knockdown was performed as described in methods and Fig. 1.Two different siRNA were used (siVRK1-02 and siVRK1-03) as well as si-Control from Dharmacon. Coilin was detected with monoclonal antibody Pdelta (Santa Cruz) and VRK1 with a policlonal antibody (Sigma). **B.** Field image obtained by confocal microscopy. **C**. Distribution (in percentage) of the quantification of CBs based on size (pixels)(top) and presence or absence of CBs (bottom) using the Image J program. Statistics were calculated with Chi-square. * ($P<0.05$)**, ($P<0.005$), ***($P<0.0005$).

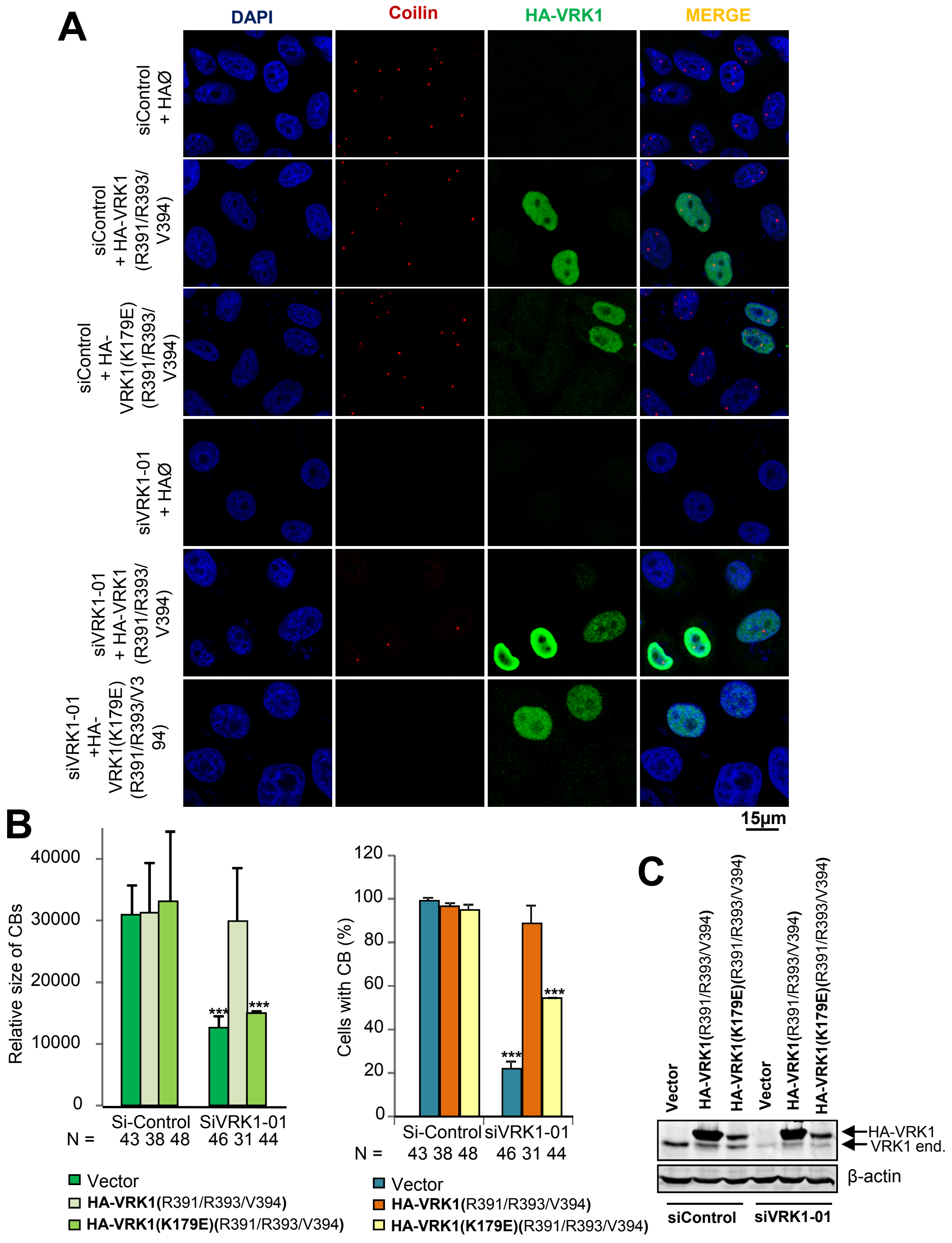

Supplementary Figure S3. Cajal bodies can be rescued by wild-type human VRK1 but not by kinase-dead VRK1. A. Rescue of defective Cajal Body formation by a siRNA-resistant VRK1. HeLa cells were transfected with siVRK1-01 to knock down endogenous VRK1, or with siControl. Cells were retransfected with plasmid HA-VRK1(R391/R393/V394), resistant to si-VRK1-01 or with the same plasmid containing the K179E substitution to make it kinase-dead. After retransfection cells were immunostained with anti-HA polyclonal antibody to identify cells expressing exogenous VRK1 active or kinase-dead. Cajal Bodies were visualized by staining with Coilin monoclonal antibody Pdelta (Santa Cruz). **B.** Quantification of the size and number of Cajal Bodies in the rescue experiments using ImageJ software (NIH). Means of the number, or size, of Cajal Bodies per cell and standard deviations are represented in the graph. The number of analysed cells is indicated below. * ($P<0.05$) ** ($P<0.005$) *** ($P<0.0005$). **C.** Efficiency of endogenous VRK1 silencing, and expression of siRNA-resistant VRK1 was determined by Western blot.

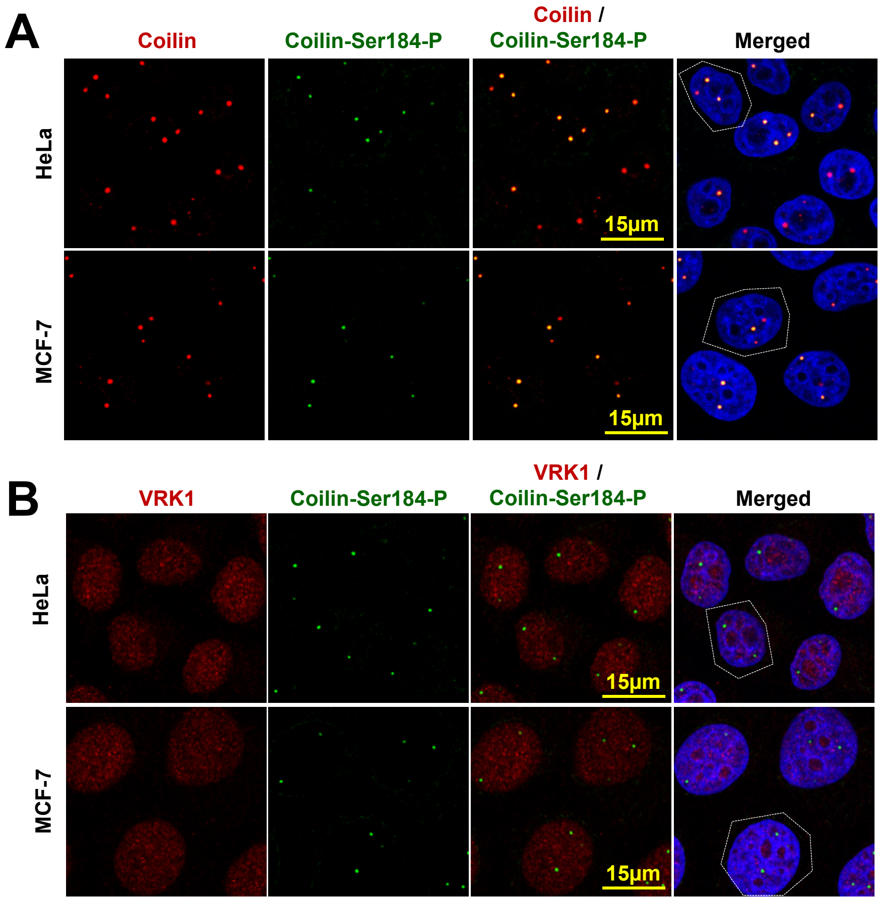

Supplementary Figure S4. **Localization of VRK1, coilin and coilin-Ser184-P.** **A.** Colocalization of coilin and coilin phosphorylated in Ser184 in MCF7 and HeLa cells. In the column right (DAPI) is marked the cell selected for scanning to show (Fig. 4a) that some CBs after assembly do not have phosphorylated coilin. **B.** Colocalization of VRK1 and coilin phosphorylated in Ser184 in MCF7 and HeLa cells. In the column right (DAPI) is marked the cell selected for scanning to show (Fig. 4b) that VRK1 is not within CBs with phosphorylated coilin. Field images corresponding to details shown in Fig. 4.

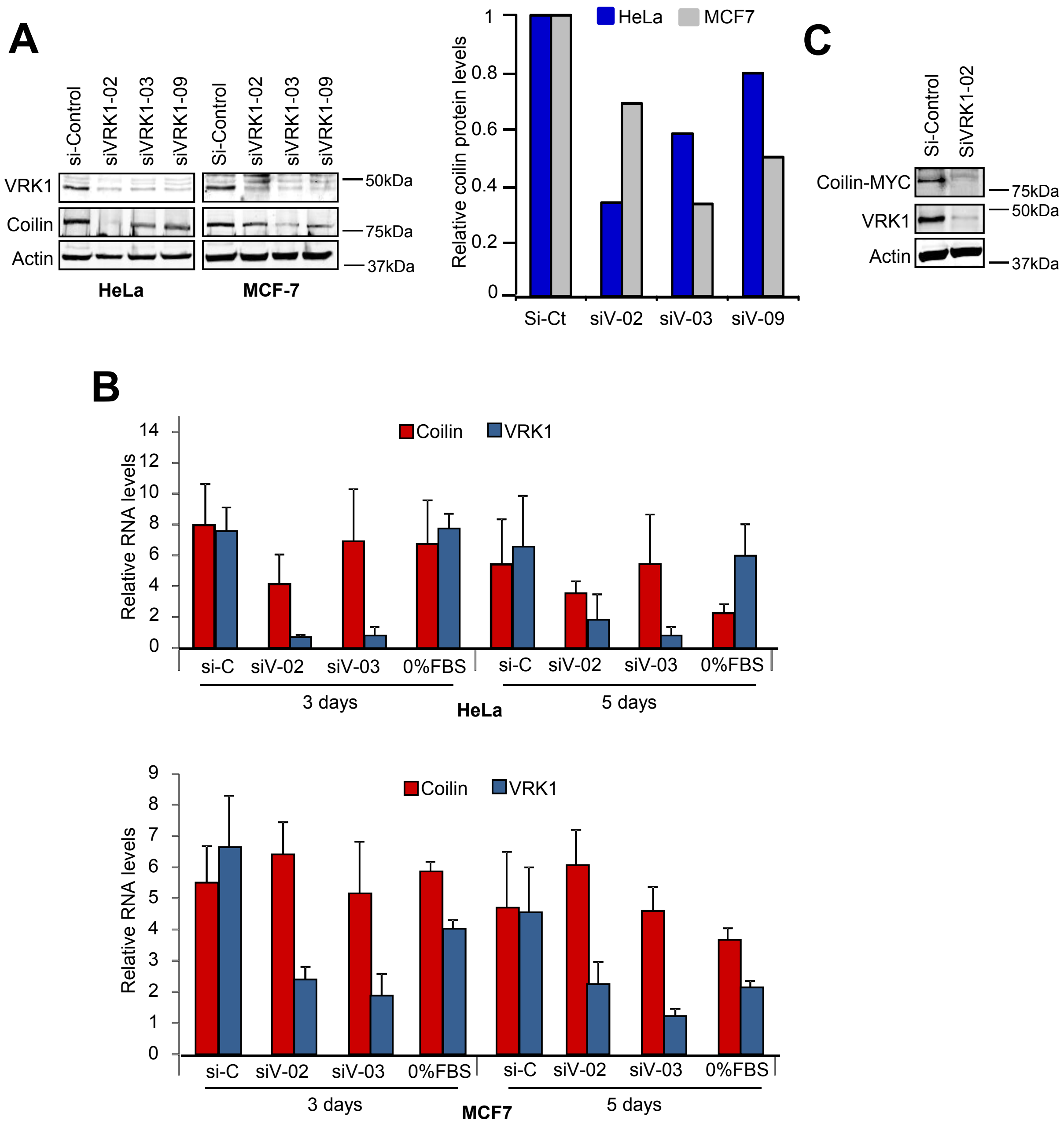

Supplementary Figure S5. VRK1 knockdown does not affect coilin gene expression. A. Effect of three different siVRK1 on the level of coilin in two cell lines, HeLa and MCF7. **B.** Effect of VRK1 knockdown on gene expression was performed in two cell lines, HeLa (top) and MCF7 (bottom), with two siVRK1 (02 and 03). Seventy-two hours after knockdown, total RNA was extracted with RNeasy Mini kit (Qiagen) and quantified in a Bioanalyzer 2100 nano-lab chip (Agilent Biotechnologies). Specific VRK1 and Coilin RNA was determined by qRT-PCR using a Quantitec SYBRgreen RT-PCR kit (Qiagen) in an iCycler thermocyler (Biorad). Values are normalized with respect to GAPDH **C.** VRK1 knockdown reduces the level of transfected coilin. VRK1 was knocked down in HeLa cells, and twenty-four hours later cells were transfected with plasmid pCMV6-Coilin-myc-DKK. Forty-eight hours after plasmid transfection the levels of endogenous VRK1 (VC antibody) and transfected coilin were determined by immunoblot.

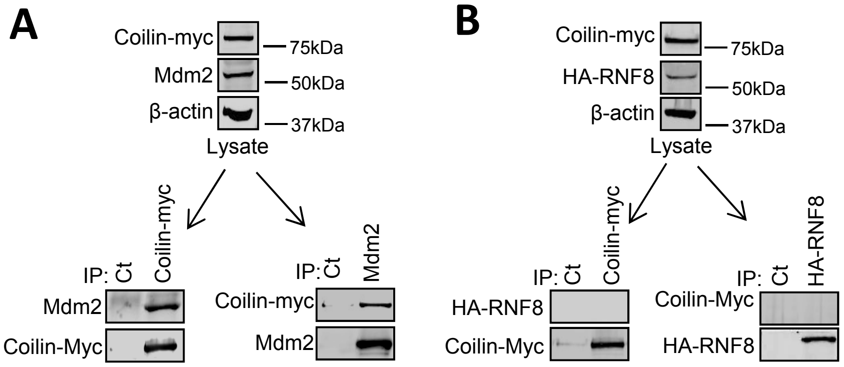

Supplementary Figure S6. Interaction of ubiquitin ligases and coilin. A. MCF7 cells were transfected with plasmids pCMV6-Coilin-myc (5 µg) and pCOC-Mdm2 (6 µg). **B.** MCF7 cells were transfected with plasmids pCMV6-Coilin-myc (5 µg) and pHA-RNF8(6 µg). The levels of the proteins were determined forty-eight hours after transfection in immunoblots. Cell lysates were used for immunoprecipitation with specific antibodies for the corresponding epitope, or mdm2.

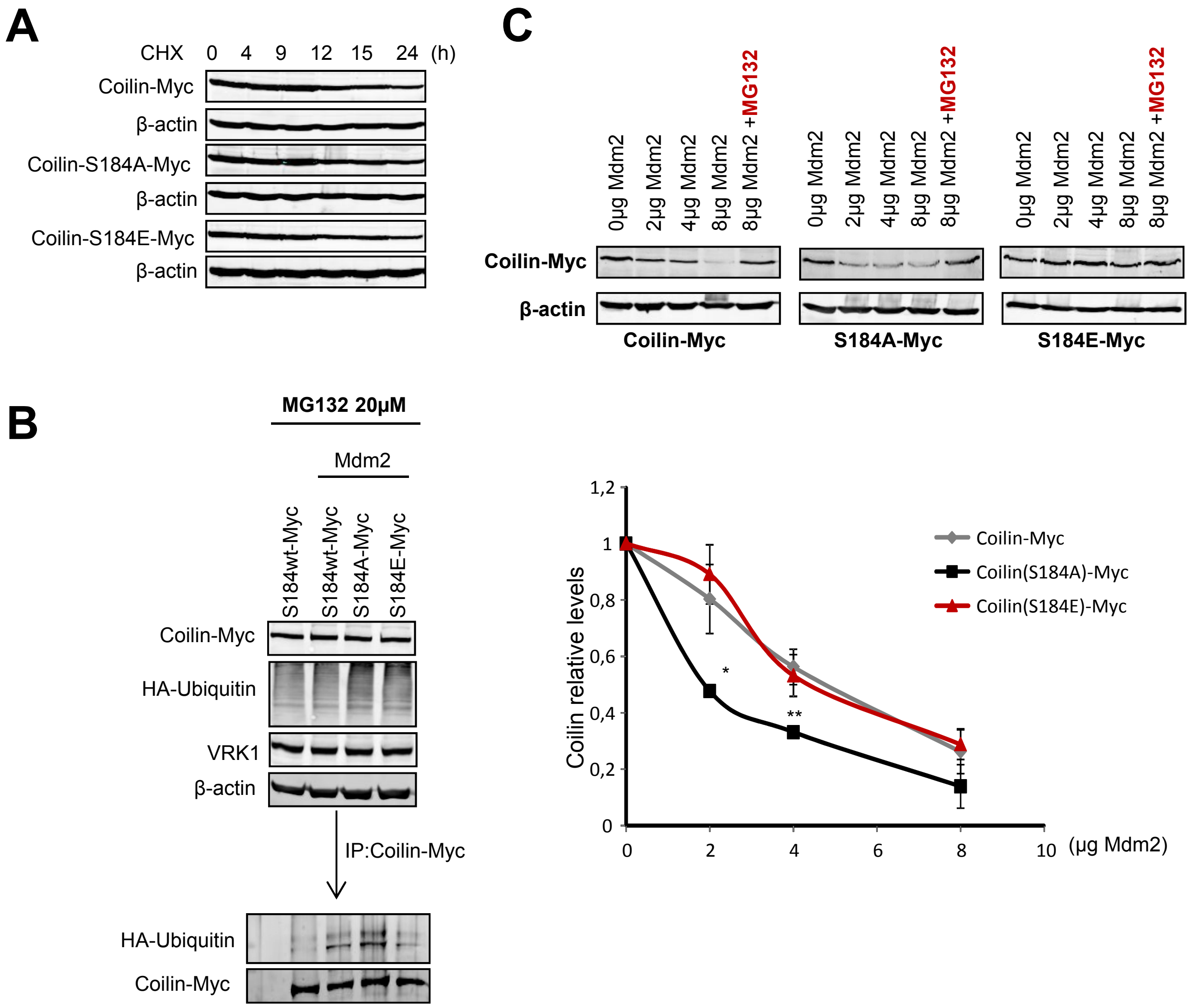

Supplementary Figure S7. Stability of coilin and its mutants in Ser184. **A.** Stability of coilin-wt or its S184A and 184D mutants. MCF7 cells were transfected with plasmids pCMV6-Coil-Myc-Flag (wild type, S184A, and S184E) y pCEFL-Ubiquitin-HA. Forty-eight hours later cycloheximide (CHX) was added to the culture and the level of transfected coilin determined at different time points. **B. Ubiquitination of coilin wild type or its Ser184 mutants.** MCF-7 cells were transfected with plasmids pCMV6-Coil-Myc-Flag (wild type, S184A y S184E) and pCEFL-Ubiquitin-HA and their expression determined forty-eight hours later. Cell lysates were incubated with the indicated antibody for six hours at 4ºC and immunoprecipitated with an anti-myc antibody. The proteins in the immunoprecipitate were detected with antibodies specific for their epitopes. **C. Sensitivity of coilin and its Ser184 phosphomutants to mdm2.** MCF-7 cells were transfected with 5 µg of plasmids pCMV6-COIL-MYC (wild type, S184A o S184E), 1 µg of plasmid pUbiquitin-His and increasing amounts of plasmid pCOC-Mdm2. The point with the highest concentration of mdm2 was also incubated with MG132 at 35uM for 6 hours. Forty-eight hours post-transfección, cells were lysed and the proteins analyzed in immunoblots. The level of coilin was quantified and represented in the graph. The values are the mean of three independent experiments. * ($P<0.05$) ** ($P<0.005$) *** ($P<0.0005$).
